# A New Risk Polymorphism rs10403848 of CARD8 Significantly Associated with Psoriasis Vulgaris in Northeastern China

**DOI:** 10.1155/2020/2867505

**Published:** 2020-02-11

**Authors:** Pei Yu, Bingmei Liu, Siyu Hao, Ronggui Xing, Yuzhen Li

**Affiliations:** ^1^Department of Dermatology, Second Affiliated Hospital of Harbin Medical University, Harbin 150081, China; ^2^Department of Dermatology, Heilongjiang Provincial Hospital, Harbin 150001, China

## Abstract

Caspase recruitment domain family member 8 (CARD8) is an adaptor molecule that negatively regulates nuclear factor-*κ*B (NF-*κ*B) activation, interleukin (IL)-1*β* secretion, and apoptosis. These play important roles in the pathogenesis of psoriasis. Genetic variants of CARD8 have been associated with an increased risk of several inflammatory diseases and psoriasis in Europe. However, nothing is known about the association of the polymorphisms of CARD8 and psoriasis vulgaris (PsV) in the Han population of northeastern China. To investigate the potential association between them, we designed a case-control study to genotype four selected single nucleotide polymorphisms (SNPs) using the improved multiplex ligation reaction (iMLDR) method. Model-based single SNP frequentist-test and haplotype association studies were performed to assess the association between SNPs and PsV. The results showed that the intron SNP rs10403848 was significantly associated with PsV (additive model *p*=0.0418, *p*′=0.0411, and statistical power 0.1902; heterozygous model *p*=0.0418, *p*′=0.0164, and statistical power 0.9406). A potential risk locus of nonsynonymous SNP rs2043211 found in the European population did not show a significant association in our study. We found that the polymorphism rs10403848 in CARD8 is significantly associated with PsV risk in the Han population of northeastern China. CARD8 may be involved in PsV in this population, as in the European population, but a different genetic process should be considered for the heterogeneity of risk loci.

## 1. Introduction

Psoriasis is an immunologically mediated chronic inflammatory and hyperproliferative skin disease affected by both genetic and environmental factors. It is characterized by epidermal hyperplasia, inflammatory cell infiltration, and vascular remodeling [1], and it affects 0.5–3% of the general population [2]. Psoriasis has been thought to be dependent on a complex interplay between many genetic loci and environmental factors [3]. The incidence of psoriasis vulgaris (PsV) accounted for more than 90%. There is abundant evidence showing that numerous risk-associated genetic variants within many susceptibility loci are related to PsV [4]. To date, people have performed numerous studies on PsV and uncovered a few novel genetic loci that are associated with the disease, including those related to innate immunity, T-cell signaling, skin barrier function, and NF-kB signaling [5]. More than 40 susceptibility genes have been identified involving diverse ethnic populations [6]. However, the SNPs identified account for only a small fraction of the hereditary factors [6], and the etiology of PsV is not fully understood. In PsV, a substantial polygenic component remains unclear but would have an impact on the development of genetic diagnosis and prognosis.

The caspase-associated recruitment domain (CARD) is a conserved homology domain and a protein-protein interaction module [7]. It has been suggested that CARD8, a member of the CARD-containing family of proteins, is a part of the inflammasome [8]. The inflammasome is a cytoplasmic protein complex that plays a vital role in the maturation and secretion of proinflammatory cytokines [8], including interleukin (IL)-1*β* and IL-18 [9–11]. These cytokines are known to cause a variety of biological effects associated with infection, inflammation, and autoimmune processes by triggering the adaptive immune response and they are strongly implicated in the pathogenesis of psoriasis [9–11]. CARD8 was shown to be a negative regulator of nuclear factor-*κ*B (NF-*κ*B) and caspase-1 activation, which implicates its role in the suppression of NLRP3 inflammasome activation, leading to the suppression of the immune response and of inflammatory activity [8]. CARD8 represents a new signaling molecule involved in the regulation of caspase-1 and NF-*κ*B activation [12], which play key roles in the development of psoriasis [13, 14].

Genetic variants of CARD8 are also important in the pathogenesis of a variety of inflammatory diseases [13]. The gene polymorphisms of CARD8 have been associated with several inflammatory and immune diseases, such as rheumatoid arthritis [15, 16], Crohn's disease [17], gout [18, 19], ankylosing spondylitis [20], and atopic dermatitis [21]. Some authors have proposed that psoriasis is associated with other autoimmune diseases, such as arthritis and Crohn's disease [22]. A polymorphism of CARD8 and rs2043211, is associated with the risk of psoriasis in Swedes [13]. The relation between CARD8 and the etiopathogenesis of PsV in the Chinese population remains unknown. Therefore, we performed a case-control study to investigate the relationship between CARD8 polymorphisms and PsV in the Han population of northeastern China.

## 2. Methods

### 2.1. Study Subjects

We randomly selected 540 PsV patients and 612 healthy controls from January 2013 to April 2015 at the Second Hospital of Harbin Medical University. We calculated the sample size to confirm the reliability of the data results. All the patients included in the study had a diagnosis of PsV as determined by two dermatologists at this institution and all had at least two skin lesions. All patients and healthy controls in this study were of Han descent of northeastern China (including three levels of relatives). The healthy controls were randomly selected from a population that came to the same hospital for health examinations. We used available self-reporting questionnaires to obtain basic information (sex and age) from both groups and clinical data (PsV severity, age of onset, and family history, including three levels of relatives) from patients. We also used the questionnaires to exclude patients and controls with any systemic, infectious, autoimmune, atopic, or malignant disease (controls were excluded if they had psoriasis or a family history of psoriasis, including three levels of relatives).

All participants provided written informed consent. Also, the study protocol was approved by the ethics committee of the Second Affiliated Hospital of Harbin Medical University. All methods in this study were conducted in accordance with the approved guidelines and the ethical guidelines of the Declaration of Helsinki.

### 2.2. Candidate SNP Selection

The method we used to identify potentially relevant SNPs is described in Zhao et al. [23]. First, we studied the length of CARD8 gene sequence. Then, we obtained genetic polymorphism data from the NCBI dbSNP database (http://www.ncbi.nlm.nih.gov/projects/SNP/). In selecting SNPs of CARD8, we considered linkage disequilibrium and different SNP distributions. Moreover, we enrolled the SNPs based on predicting the potential function in the predisposition to PsV in the GWAS study and referring to the related researches. Finally, we selected four representative common SNPs, rs2043211, rs2288876, rs3745718, and rs10403848, for which the Minor Allele Frequency (MAF) was greater than 0.05 in the HapMap Chinese Han Beijing population. The selection of the CARD8 polymorphism rs2043211, a missense locus, was based on its previous association with autoinflammatory diseases [13, 15–22]. rs2288876 is a synonymous polymorphism, rs3745718 locates on the 3′-untranslated regions, and rs10403848 locates to an intron region.

### 2.3. DNA Extraction and Genotyping

Venous whole blood samples (2 ml each) were collected from each participant in tubes containing ethylenediamine tetra-acetic acid and stored at −20°C according to the manufacturer's recommendations. Genomic DNA was extracted using a QIAamp DNA blood Mini Kit (Qiagen, Germany), according to the protocol of the manufacturer. The target DNA sequences were amplified using a multiplex polymerase chain reaction (PCR) method with specific primers. The primer DNA sequences are shown in Tables 1-2. All the genotyping experiments were performed using the iMLDR as described by Genesky Biotechnologies Inc. (Shanghai, China) [23].

The PCR was carried out on the ABI 2720 Thermal Cycler (Applied Biosystems, USA) in a 20 *μ*l reaction mixture (total volume). The ABI3730XL sequencer was used for genotype detection, and the results were analyzed with GeneMapper 4.1 software (Applied Biosystems, USA). The staff who performed the genotyping were blinded to the sample groups. To verify reproducibility, 5% of the samples were selected at random for a repeat PCR.

### 2.4. Allele, Genotype, and Haplotype Association Studies

The demographic characteristics were analyzed using SPSS version 19.0 (SPSS, Inc., Chicago, IL, USA). The distribution description of detected genotypes and alleles and the Hardy–Weinberg equilibrium (HWE) test were performed using Haploview 4.0 (http://www.broad.mit.edu/mpg/haploview/); the HWE test threshold was defined as 0.001. We defined the polymorphism effect with codominant (allele), additive (genotype), dominant, recessive, and heterozygous models based on the allele frequencies of each locus in the PsV cases. The allele and genotype frequencies were statistically compared in the cases and controls. The chi-square test was used to perform association studies of the CARD8 polymorphisms between patients and controls. The single SNP association studies were completed by the SNP test. Statistical significance was defined as *p* < 0.05 for a single test, and 10,000 permutations were performed for the multiple test correction. The linkage disequilibrium (LD), haplotype inference, and association study were performed by Haploview. The LD *r*^2^ was limited to 0.8, and *p* < 0.05 was considered statistically significant.

## 3. Results

### 3.1. Samples and Genotyping Data Description

The study included 1152 persons who were genotyped. The proportion of males in both groups was higher than that of females (124 more in the case group and 72 more in the control group), which may be due to the selection bias. There were no significant differences in the distribution of sex and age between the two groups (*p*=0.20 and *p*=0.24, separately). We stratified and compared the PsV patients according to sex, age at disease onset (early onset (≤40 years) and late onset (>40 years)), severity index (severe (PASI > 10) and mild (PASI < 10)), and family history. However, we did not find any evidence that these factors were associated with PsV risk. As an indicator of quality control for the genotyping test, the blinded genotyping concordance rate was 100%, and no discrepancies were identified in the randomly selected 5% (58) of samples that were subject to repeat PCR. The basic demographic and clinical data for the participants are shown in Table 3.

In 1152 quality control samples, the genotype recall rate was 100%. The genotype frequencies of CARD8 polymorphisms (rs2043211, rs2288876, rs3745718, and rs10403848) in the study were common SNPs (MAF > 0.05), as estimated. In both the case and control groups, none of the studied polymorphisms deviated from HWE, and no SNP demonstrated a Mendelian genetic error.

### 3.2. Single SNP Association Studies

For the rs10403848 SNP, the minor A allele (risk allele) was present in 30.7% of patients and 28.9% of controls. The additive model (G vs. A) distribution was significantly different between cases and controls (*p*=0.0418, *p*′=0.0411), and the heterozygous model (AA + GG vs. AG) distribution was significantly different between cases and controls (*p*=0.0164, *p*′=0.0159). We calculated the power of the sample using the PASS11, and we found that the power of the additive model distribution was 0.1902 and the heterozygous model distribution was 0.9406, which indicated that CARD8 may be associated with the development of PsV. It was determined that the G allele and G-carriers of rs10403848 correlated negatively with the susceptibility to PsV from the additive model, whereas the heterozygous AA + GG genotype had a protective effect from the heterozygous model. When comparing genotype distributions of the remaining three SNPs (rs2043211, rs2288876 and rs3745718) between cases and controls, no significant differences were demonstrated. These are listed in Tables 4-5.

### 3.3. Haplotype-Based Association Studies

We performed haplotype analysis to evaluate the combined effects of the four SNPs on PsV risk. Among all the haplotypes inferred by Haploview, the frequency of eight haplotypes exceeded 0.01. Haplotype frequencies and association study results are shown in Table 6. Among the eight haplotypes, the most common were TTAA and TTAG, the frequencies of which were 0.250/0.230 (*χ*^2^ = 0.625, *p*=0.4292) and 0.219/0.2340 (*χ*^2^ = 0.395, *p*=0.5294), respectively. We did not find any of the eight genotypes significantly associated with PsV risk (*p* > 0.05).

## 4. Discussion

In this case-control study, we investigated CARD8 polymorphisms and clinical parameters as possible risk factors for PsV in northeastern China. We assessed the contribution of CARD8 (rs2043211, rs2288876, rs3745718, and rs10403848), and we found that two genotypes of rs10403848 were associated with PsV risk. This finding may provide new information suggesting the importance of the CARD8 gene in PsV.

**Figure 1:**
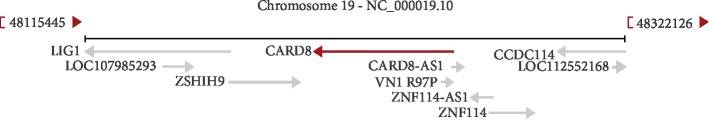
Schematic diagram of CARD8 gene location.

**Figure 2:**
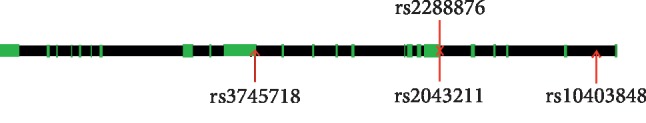
Schematic diagram of CARD8 gene structure and SNP location.

Identifying susceptible genes is expected to elucidate the etiology of PsV and guide clinical treatment. Incomplete coverage of a genome-wide association study (GWAS) may produce unsatisfactory results. In addition, psoriasis has characteristics affected by both genetic and environmental factors, which made it necessary to test the association of different regions of genes for suspected SNPs. CARD is a conserved homology domain and a protein-protein interaction module that plays important roles in apoptosis, the NF-*κ*B signaling pathway, and cytokine regulation. CARD8 gene is located on chromosome 19 (19q13.33), NC_000019.10 (48203148..48256264, complement), as shown in Figure 1. In the gene schematic diagram (Figure 2), we can see the gene contains 22 exons (the green ones are exons, and the parts between the green ones are introns). The position of this gene is from left to right. rs10403848 is not in the first intron, but in the last intron. CARD8 is just one gene and contains no other genes. The polymorphism of CARD8 gene impacts its expression levels, and there is a large body of literature on CARD8 SNPs and their correlation with inflammasome protein expression and activation [24]. rs2043211 is the most studied susceptibility locus. It showed a significant correlation with psoriasis risk in a Swiss study [13]. It also has been associated with susceptibility to Crohn's disease, a disease that shares several susceptibility loci with psoriasis [25]. However, no significant correlation was found in our study. We did find a significant association between rs10403848 and the risk of PsV. Zhao et al. studied the same SNP in patients with prehypertension and coronary atherosclerosis, which are also inflammation-related diseases but did not find a significant difference between patients and controls. Our different findings may suggest the heterogeneity of different populations (including ethnic origin, eating habits, environmental exposure, health habits, and so on) and the variability of different diseases. In addition, in our research, the heterozygous model received a lower *p* value than the additive model. It seemed that the risk allele was AG allele in the heterozygous model rather than A allele in the additive model, and the heterozygous model was the best association model compared with other models. However, this phenomenon has not been reported in other PsV association studies. We found that the results were different in different studies. Therefore, we could not infer that the heterozygous model is the best association model for all known associated sites. Maybe we need more research to explain it in the future. Potential functional annotation of rs10403848 was identified using the HaploReg version 4.1database (http://www.broadinstitute.org/mammals/haploreg), and the application of this method is modeled on some previous studies on psoriasis [26]. According to the Haploreg dataset, we found that rs10403848 is a trans eQTL for *CARD8* in whole blood suggesting that the effects of the rs10754557 and CARD8 expression may differ in disease developing risk for individuals with the different genetic genotype. To a certain extent, this also explained the reasons for the different results of the abovementioned studies. Available Haploreg data also indicated that rs10403848 was located in the intron region, and Histone marks (H3K4me1_Enh) were detected at the locus based on histone modification feature data in many immune-related cell lines, such as Primary T helper memory cells from peripheral blood, Primary T CD8+ naive cells from peripheral blood, and Primary monocytes from peripheral blood, suggesting that this locus may function as an enhancer in an immune-related system.

CARD8 interacts with leucine-rich repeat and pyrin domain containing 3 (NLRP3) and Apoptosis-associated Speck-like protein containing a CARD to form a caspase 1 activating complex termed the NLRP3 inflammasome [26]. As we mentioned earlier, the NLRP3 inflammasome is closely related to the mechanism of psoriasis. Moreover, we have previously discovered the relationship between the genetic polymorphism of NLRP3 (rs3806265 and rs10754557) and psoriasis. In this experiment, we found a new susceptible site of CARD8. These findings underscore the important role of autoimmunity and inflammation in the development of psoriasis. It also gives us a new understanding of this relationship and provides us with more perspectives on the study of psoriasis.

The etiopathogenesis of PsV is multigenic and multifactorial. Like our previous studies, [27] our study had several limitations. First, our sample size was relatively small, which may limit the revelation of true relationships. An increase in the sample size may reveal an even more pronounced significance. Second, all of our samples were from the Han population of northeastern China. This restriction to a single population and region might introduce a selection bias, and our results may not apply to other ethnic groups. In addition, genes and environment interact with each other, which may play a part in the pathogenesis of psoriasis, so different environmental risk factors in different populations may also affect the role of susceptibility loci in psoriasis risk. Third, we only tested limited polymorphisms in the CARD8 gene, which may not be a critical locus for psoriasis but just near in LD to a causative locus. Thus, studies on the correlation of more gene loci and the functional role of genes are needed. In spite of these limitations, our study demonstrated an association between the rs10403848 polymorphism and the risk of PsV. This result could provide reference information for subsequent functional studies.

## 5. Conclusion

In conclusion, our study suggested a correlation between the variant SNP rs10403848 of the CARD8 gene and PsV in the Han population of northeastern China. *CARD8* may be involved in psoriasis development as a genetic marker or a causative polymorphism. Previously, our team identified a relationship between several other genetic polymorphisms and PsV [14, 27–29]. We will compile these findings to further explore the relationship between these genes and PsV in the Han population of northeastern China. At the same time, large-scale, multiple gene studies in ethnically diverse populations will be necessary.

## Figures and Tables

**Table 1:** Primers used for the genotyping of CARD8.

SNP	PCR primer (5′-3′)	PCR primer (3′-5′)	Product length (bp)
rs2043211	TGTCCCCCCCAGATAGTTGACA	GATGGAGTCGTAGGGGCCTGAG	281
rs2288876	TGTCCCCCCCAGATAGTTGACA	GATGGAGTCGTAGGGGCCTGAG	281
rs3745718	TGCTCAGCAAGGCCTCATTCTT	GGTGCAGCCTTTGTGAAGGA GA	166
rs10403848	GCCACTTCCCCACCCCTCAT	TGCACCTGCTCCCCATTGATTA	221

SNP, single nucleotide polymorphism; PCR, polymerase chain reaction.

**Table 2:** iMLDR probe sequences.

SNP allele	Primer (5′-3′)	LDR product
rs2043211_modify	CMATAATGGCTCTGCCTCTRTCTCTTTTTTTTTTTTTTTTTT	63.56
rs2043211_A	TTCCGCGTTCGGACTGATATTGACACTCAGGAACAGCACGCAA	67.48
rs2043211_T	TACGGTTATTCGGGCTCCTGTTGACACTCAGGAACAGCACGCAT	66.45
rs2288876_modify	TGTGACATCTCACATTTTTTCCAAGATTTTTTTTTTTTTTTTTTTTTTTT	63.96
rs2288876_A	TACGGTTATTCGGGCTCCTGTGAGCTACCCTGTGTTTCTGAGACCGTT	66.99
rs2288876_G	TTCCGCGTTCGGACTGATATGAGCTACCCTGTGTTTCTGAGACCATC	67.53
rs3745718_modify	GACAATGAGGTTCTTACTGAGAATGAGAATTTTTTTTTTTTTTTTTTTTT	63.30
rs3745718_C	TCTCTCGGGTCAATTCGTCCTTGGGGTGCTyGATGATCTCAAG	66.47
rs3745718_T	TGTTCGTGGGCCGGATTAGTGGGGTGCTyGATGATCTCGAA	66.80
rs10403848_modify	ATTCATATTGTTGCTCCAGATTCTTTTTAATTTTTTTTTTTTTTTTTTTTTTTTTT	63.28
rs10403848_A	TGTTCGTGGGCCGGATTAGTGAAATGCTCTTGAAGCCTAATTTGTCTCAT	66.49
rs10403848_G	TCTCTCGGGTCAATTCGTCCTTGAAATGCTCTTGAAGCCTAATTTGTCTCAC	66.96

SNP, single nucleotide polymorphism; LDR, multiplex ligation detection reaction.

**Table 3:** The basic demographic and clinical data for the participants.

Characteristic	Cases	Controls	*p* Value
Total number	540	612	
Gender, *n* (%)			0.062
Male	332 (0.615)	342 (0.559)	
Female	208 (0.385)	270 (0.441)	
Mean age ± SD, years	44.23 ± 12.44	45.47 ± 12.73	0.238
PASI, *n* (%)			
≤10	446 (0.826)		
>10	94 (0.174)		
*p* Value	0.127		
Age at onset, *n* (%)			
≤40 years	439 (0.813)		
>40 years	101 (0.187)		
*p* Value	0.201		
Family history, *n* (%)			
Yes	203 (0.376)		
No	337 (0.624)		
*p* Value	0.122		

**Table 4:** Candidate SNPs analysis.

Gene	SNP	Chromosome position	Major/minor allele	Risk allele	Risk allele frequency
					Case control
*CADR8*	rs2043211	48234449	A/T	A	0.520, 0.518
	rs2288876	48234507	A/G	G	0.259, 0.243
	rs3745718	48211896	T/C	T	0.746, 0.745
	rs10403848	48253518	G/A	A	0.307, 0.289

SNP, single nucleotide polymorphism.

**Table 5:** The single SNP association studies result of CARD8 for the four variants in cases and controls.

SNP	Genotype	Cases (*n* = 540)	Controls (*n* = 612)	H-W *P*	Statistical model	*p*	*p*′	OR (95%CI)
rs2043211	TT	118 (0.219)	140 (0.229)		Codominant	0.9343	0.9491	
	TA	282 (0.522)	310 (0.506)		Additive	0.9268	0.9284	1.010
	AA	140 (0.259)	162 (0.265)	0.8727	Dominant	0.7686	0.8411	(0.801, 1.273)
	T/A	518/562	590/634		Recessive	0.8821	0.9237	
		0.480/0.520	0.482/0.518		Heterozygous	0.7070	0.7328	
rs2288876	GG	30 (0.056)	30 (0.049)		Codominant	0.5251	0.5328	
	GA	220 (0.407)	238 (0.389)		Additive	0.8168	0.8158	0.919
	AA	290 (0.537)	344 (0.562)	0.6794	Dominant	0.7249	0.8551	(0.704, 1.201)
	G/A	280/800	298/926		Recessive	0.5464	0.5559	
		0.259/0.741	0.243/0.757		Heterozygous	0.6505	0.6676	
rs3745718	TT	300 (0.556)	328 (0.536)		Codominant	0.9617	0.9999	
	TC	206 (0.381)	256 (0.418)		Additive	0.5029	0.5123	0.994
	CC	34 (0.063)	28 (0.046)	0.5818	Dominant	0.6372	0.6758	(0.762, 1.296)
	T/C	806/274	912/312		Recessive	0.3615	0.4544	
		0.746/0.254	0.745/0.255		Heterozygous	0.3681	0.3930	
rs10403848	GG	244 (0.452)	318 (0.520)		Codominant	0.4950	0.5089	
	GA	260 (0.481)	234 (0.382)		Additive	0.0418^*∗*^	0.0411^*∗*^	1.091
	AA	36 (0.067)	60 (0.098)	0.3829	Dominant	0.1043	0.1080	(0.847, 1.405)
	G/A	748/332	870/354		Recessive	0.1713	0.1800	
		0.693/0.307	0.711/0.289		Heterozygous	0.0164^*∗*^	0.0159^*∗*^	

H-W, Hardy–Weinberg equilibrium test; *p*, *p* value; *p*′, *p* value adjusted by permutation; OR, odds ratio; 95% CI, 95% confidence interval, ^∗^the significant association.

**Table 6:** The haplotype-based association study of CARD8 gene polymorphisms.

Haplotype	Freq.	Case, control ratio counts	Case freq,control freq	χ^2^	*p*
TTAA	0.24	270.4 : 809.6, 282.2 : 941.8	0.250, 0.230	0.625	0.4292
TTAG	0.227	236.2 : 843.8, 286.6 : 937.4	0.219, 0.234	0.395	0.5294
TAAG	0.188	189.8 : 890.2, 243.8 : 980.2	0.176, 0.199	1.028	0.3107
CAGG	0.178	194.8 : 885.2, 215.6 : 1008.4	0.180, 0.176	0.033	0.8558
TAGG	0.056	70.8 : 1009.2, 58.0 : 1166.0	0.065, 0.047	1.791	0.1808
CAAG	0.044	50.6 : 1029.4, 51.8 : 1172.2	0.047, 0.042	0.143	0.7056
TAAA	0.031	35.4 : 1044.6, 36.4 : 1187.6	0.033, 0.030	0.089	0.7656
CAGA	0.011	10.8 : 1069.2, 15.6 : 1208.4	0.010, 0.013	0.203	0.6523

Freq, frequency; χ^2^, chi square; P, *p* value.

## Data Availability

The SNPs-related data used to support the findings of this study are available from the corresponding author upon request.

## Abbreviations

SNP: Single nucleotide polymorphism
PsV: Psoriasis vulgaris
CARD8: Caspase recruitment domain family member 8
NF- *Κ*b: Nuclear factor- *Κ*b
IL: Interleukin
iMLDR: The improved multiplex ligation detection reaction
PCR: Polymerase chain reaction
HWE: Hardy–Weinberg equilibrium
GWAS: Genome-wide association study
NLRP3: Leucine-rich repeat and pyrin domain containing 3.
